# Beyond VISION 2020: universal eye health coverage and the elimination of trachoma

**Published:** 2019-12-17

**Authors:** Peter Holland, Serge Resnikoff

**Affiliations:** 1 Chief Executive: International Agency for the Prevention of Blindness, London, UK.; 2 Immediate Past Chair: International Coalition for Trachoma Control, Lisbon, Portugal.

**The WHO World Report on Vision provides a strategic path to achieve sustainable eye health systems and universal eye health coverage.**

**Figure:**
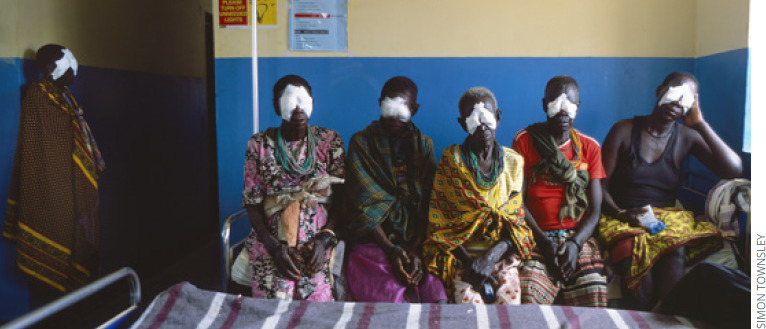
Women in Napak, Uganda, after trichiasis surgery at a camp set up by the local health department in collaboration with Sightsavers as part of The Queen Elizabeth Diamond Jubilee Trust Trachoma Initiative, with support from UK Aid.

On 18 February 1999, the World Health Organization (WHO) and the International Agency for the Prevention of Blindness (IAPB) launched **VISION 2020: The Right to Sight**. This global initiative was created to eliminate causes of avoidable blindness by the year 2020.

VISION 2020 aims to build comprehensive and sustainable eye health systems by integrating existing health services and ensuring high quality universal eye care. VISION 2020 has three key objectives: 1) the control of diseases that affect eye health; 2) the development of human resources; and 3) the provision of appropriate technology and infrastructure.

Trachoma, the world's leading infectious cause of blindness, is one of the priority diseases targeted by VISION 2020. There has been significant advancement towards elimination since the launch of VISION 2020. In June 2019, **WHO announced a 91% global reduction** in the number of people at risk of trachoma, from 1.5 billion in 2002 to 142.2 million today. During the same period, the number of people requiring surgery for trachomatous trichiasis (TT), the late blinding stage of trachoma, reduced from 7.6 million to 2.5 million – a 68% reduction. Nine countries across all endemic WHO regions have also been validated for achieving elimination.

Progress towards VISION 2020's mission and objectives include:

1. **Raising support for comprehensive and sustainable eye health systems**
  VISION 2020 has led several initiatives that have raised the profile of avoidable blindness. Four World Health Assembly (WHA) resolutions have been adopted since 2003, including WHA resolution 66.4 **Universal Eye Health: A global action plan 2014 – 2019**, which have reinforced WHA Resolution 51:11 calling for the global elimination of trachoma. WHA resolutions aim to support UN member states to achieve global vision targets, including universal access to comprehensive eye care services.
2. **Robust evidence to support disease elimination efforts**
  International commitments and increased investment led to the largest ever infectious disease survey – the **Global Trachoma Mapping Project** (GTMP). From 2012–2016, GTMP screened over 2.6 million people for trachoma across 29 countries and identified areas where interventions needed to be scaled up. Since 2016, **Tropical Data** has supported health ministries through the full survey process - from planning and protocol development to application of the survey outputs. Data collected by GTMP and Tropical Data have mobilised resources to scale up all components of the WHO-endorsed SAFE strategy (surgery, antibiotics, facial cleanliness, environmental improvements) and have contributed to **over 566 million doses of antibiotics being distributed and nearly 1.5 million TT operations being conducted** since 2011.
3. **Improved human resources, infrastructure and technology for eye health**
  In recent years, programmes have included strategies to **effectively use limited human resources** in resource-poor settings.^1^ In Kenya, Tanzania and Chad, national programmes are upskilling ophthalmic nurses and ophthalmic clinical officers to carry out and manage TT operations. In Ethiopia, **which accounts for 44% of the global burden of trachoma**, the national programme is training general health workers to provide eye care services, including TT surgery, in order to improve coverage rates. Furthermore, new innovations, such as the TT tracker, is **helping national programmes to track surgical performance** for individual surgeons, so supervisors know when enhanced supervision or additional training is needed.^2^

## Conclusion

With VISION 2020 coming to an end next year, the eye health sector can celebrate advancements towards building sustainable eye health systems. The WHO World Report on Vision, published in October 2019, provides a strategic path to progress towards objectives set by VISION 2020. However, to achieve targets, trachoma interventions must be included in national eye health care plans and health systems must be equipped to deliver comprehensive eye health care for entire populations, including people with disabilities and other hard to reach populations.

## References

[1] Courtright P Jesudason T Lewellan S. Achieving universal eye health coverage: planning and human resource lessons from trachoma. Comm Eye Health Vol. 31 No. 102 2018 pp 54. PMC6134455 30220811

[2] Bartlett S Jensen K Jesudason T. TT Tracker app aims to improve surgical outcomes and patient care. Comm Eye Health Vol. 31 No. 104 2019 pp 93 PMC6390511 31086441

